# Heavy Metal Resistance Genes Are Associated with *bla*_NDM-1_- and *bla*_CTX-M-15_-Carrying Enterobacteriaceae

**DOI:** 10.1128/AAC.02642-17

**Published:** 2018-04-26

**Authors:** Qiu E. Yang, Siham Rajab Agouri, Jonathan Mark Tyrrell, Timothy Rutland Walsh

**Affiliations:** a Department of Medical Microbiology and Infectious Disease, Division of Infection and Immunity, Cardiff University, Cardiff, United Kingdom

**Keywords:** heavy metal resistance, *bla*_NDM-1_, *bla*_CTX-M-15_, plasmids, coresistance

## Abstract

The occurrence of heavy metal resistance genes in multiresistant Enterobacteriaceae possessing *bla*_NDM-1_ or *bla*_CTX-M-15_ genes was examined by PCR and pulsed-field gel electrophoresis with S1 nuclease. Compared with clinical susceptible isolates (10.0% to 30.0%), the *pcoA*, *merA*, *silC*, and *arsA* genes occurred with higher frequencies in *bla*_NDM-1_-positive (48.8% to 71.8%) and *bla*_CTX-M-15_-positive (19.4% to 52.8%) isolates, and they were mostly located on plasmids. Given the high association of metal resistance genes with multidrug-resistant Enterobacteriaceae, increased vigilance needs to be taken with the use of heavy metals in hospitals and the environment.

## TEXT

The increasing spread of multidrug-resistant superbugs in clinical environments has prompted worldwide concern, because antibiotic resistance genes, such as *bla*_NDM-1_ and *bla*_CTX-M-15_, limit treatment options to combat bacterial infections (1–4). Note that in addition to emerging antibiotic resistance, heavy metals represent another major source of environmental contamination that may select for antibiotic resistance (5). Heavy metal compounds for growth promotion and therapeutic treatment, like zinc and copper, have been used in pig and poultry production; and unlike antibiotic food additives, metals can accumulate in soil, water, aquacultural and marine antifouling treatments, and industrial effluent (6). It has been proposed that antibiotic-resistant bacteria are enriched at locations contaminated with metals, and genes conferring coselection to heavy metals and antibiotics are often found together in many clinical isolates (7–11). Furthermore, genes conferring heavy metal tolerance may coexist on the same genetic element (e.g., plasmid), which may further promote codissemination and resistance (10, 12). Here, we characterize the phenotype and genotype of heavy metal resistance in a collection of clinical Gram-negative isolates, including Klebsiella pneumoniae, Escherichia coli, Enterobacter cloacae, Klebsiella oxytoca, and Providencia stuanti, isolated from the United Kingdom and India.

A total of 95 nonduplicate isolates were tested in this study (Table 1): 39 *bla*_NDM-1_-positive isolates originated from human lower respiratory and urinary tract samples from the United Kingdom and Chennai and Haryana, India, as previously described (13); 36 *bla*_CTX-M-15_-carrying isolates originated from patients with burns, bacteremia, and urinary tract infections (UTIs) from various Indian hospitals (Haryana, Mumbai, Kolkata, Kerala, Delhi, and Vellore); and 20 control E. coli and K. pneumoniae isolates susceptible to all known antibiotic classes as control samples were provided by Specialist Antimicrobial Chemotherapy Unit (SACU), Public Health Wales. MICs of four heavy metal ions, i.e., CuSO_4_.5H_2_O for copper (Cu^2+^), HgCl_2_ for mercury (Hg^2+^), AgNO_3_ for silver (Ag^+^), and AsNaO_2_ for arsenic (As^3+^), were measured by agar dilution using Mueller-Hinton agar (Becton Dickinson, USA). E. coli (ATCC 25922) was used as a negative control. MIC levels of ≥10 mM for Cu^2+^, ≥2 mM for As^3+^, ≥32 μM for Hg^2+^, and ≥ for 128 μM Ag^+^ were regarded as resistance (8, 14, 15). High MIC values for Cu^2+^ (10 mM), As^3+^ (20 mM), and Hg^2+^ (128 μM) were obtained in most of the *bla*_NDM-1_-positive isolates, with high resistance rates of 79.5% (31/39), 76.9% (30/39), and 64.1% (25/39), respectively. Similarly, with *bla*_CTX-M-15_-positive strains, 91.7% (33/36), 63.9% (23/36), and 52.8% (19/36) of isolates were resistant to Cu^2+^, As^3+^, and Hg^2+^, respectively. High MIC values (128 to 256 μM) for Ag^+^ were observed for all isolates. Antibiotic-susceptible control strains also gave high rates of resistance to Cu^2+^ (90% [18/20]) but remained sensitive to Hg^2+^ (15.0% [3/20]) and As^3+^ (25.0% [5/20]).

**TABLE 1:** Phenotypic and genotypic resistances to heavy metals in 95 clinical strains in this study

Strain and identification no.	Bacterial organism	Phenotype (MIC)				Genotype
		Ag (μM)	Hg (μM)	Cu (mM)	As (mM)	
*bla*_NDM-1_ (*n* = 39)						
N1	K. pneumoniae	128	128	10	0.625	*merA*, *silC*
N2	K. pneumoniae	128	128	10	2.5	*arsA*, *merA*
N3	C. freundii	128	128	10	2.5	*arsA*, *merA*
N4	E. cloacae	128	16	10	20	*pcoA*, *silC*
N5	Enterobacter spp.	128	16	5	1.25	Negative
N6	E. coli	128	128	10	20	*arsA*, *merA*, *pcoA*, *silC*
N7	K. pneumoniae	128	128	10	10	*arsA*, *merA*, *pcoA*, *silC*
N8	K. pneumoniae	128	128	10	20	*arsA*, *merA*, *pcoA*, *silC*
N9	K. pneumoniae	128	16	10	0.625	*pcoA*, *silC*
N10	K. pneumoniae	128	16	10	0.625	*silC*
N11	K. pneumoniae	128	16	10	0.625	*silC*
N12	K. pneumoniae	256	128	10	10	*arsA*, *merA*, *pcoA*, *silC*
N13	C. freundii	256	128	10	10	*arsA*, *merA*, *pcoA*, *silC*
N14	E. coli	128	128	10	10	*arsA*, *merA*, *pcoA*, *silC*
N15	E. coli	128	16	5	1.25	*pcoA*, *silC*
N16	K. pneumoniae	128	128	10	1.25	*arsA*, *merA*, *pcoA*,*silC*
N17	K. pneumoniae	128	128	10	20	*arsA*, *merA*, *pcoA*, *silC*
N18	K. pneumoniae	128	64	10	10	*arsA*, *merA*, *pcoA*, *silC*
N19	K. pneumoniae	128	128	10	20	*arsA*, *merA*, *pcoA*, *silC*
N20	E. coli	128	16	5	2.5	Negative
N21	K. pneumoniae	128	128	10	2.5	*merA*, *pcoA*, *silC*
N22	K. pneumoniae	128	128	10	2.5	*merA*, *pcoA*, *silC*
N23	E. coli	128	128	5	0.625	Negative
N26	Enterobacter spp.	128	128	10	10	*arsA*, *merA*, *pcoA*
N27	K. pneumoniae	128	128	5	10	*arsA*, *merA*, *pcoA*, *silC*
N28	K. oxytoca	128	16	10	5	*arsA*, *merA*, *pcoA*, *silC*
N29	E. coli	128	16	10	10	*arsA*, *silC*
N31	E. cloacae	128	16	10	20	*pcoA*, *arsA*, *silC*
N32	E. cloacae	128	16	10	0.625	*pcoA*, *silC*, *merA*, *arsA*
K15	K. pneumoniae	128	16	10	5	*merA*, *pcoA*, *silC*
K7	K. pneumoniae	128	128	10	2.5	*merA*, *pcoA*, *silC*
IR25	K. pneumoniae	128	128	10	5	*merA*
IR18k	K. pneumoniae	128	128	10	20	*merA*
IR28k	K. pneumoniae	128	128	10	20	*merA*, *pcoA*, *silC*
IR29	E. coli	128	128	5	5	*merA*, *pcoA*, *silC*
IR26	E. coli	128	128	5	5	Negative
IR22	E. coli	128	16	5	5	Negative
IR61	K. oxytoca	128	16	10	20	Negative
IR5	E. coli	128	128	10	20	*arsA*, *merA*, *pcoA*, *silC*
*bla*_CTX-M-15_ (*n* = 36)						
A5/3	K. pneumoniae	128	16	10	5	*arsA*, *pcoA*, *silC*
A5/7	K. pneumoniae	128	128	10	20	*arsA*, *merA*, *pcoA*, *silC*
A5/4	K. pneumoniae	128	128	5	5	*pcoA*, *silC*
C5/8	K. pneumoniae	128	64	10	0.625	*arsA*, *merA*
C5/7	K. pneumoniae	128	128	10	10	*arsA*, *merA*, *pcoA*, *silC*
C5/5	K. pneumoniae	128	16	10	5	Negative
D5/12	K. pneumoniae	128	128	10	0.15	*merA*
D5/4	K. pneumoniae	128	16	10	0.625	*pcoA*, *arsA*
E5/14	K. pneumoniae	128	16	10	5	*merA*, *pcoA*, *silC*
E5/17	K. pneumoniae	128	128	10	2.5	*arsA*, *merA*, *pcoA*, *silC*
G5/2	K. pneumoniae	128	16	10	5	*arsA*, *pcoA*, *silC*
G5/6	K. pneumoniae	128	128	10	0.3	*merA*
G5/11	K. pneumoniae	128	128	10	0.3	*merA*, *pcoA*, *silC*
I5/5	K. pneumoniae	128	128	10	20	*merA*, *pcoA*, *silC*
F5/6	K. pneumoniae	128	16	10	0.3	Negative
E5/19	K. pneumoniae	128	128	10	5	*merA*, *pcoA*, *silC*
A4/8	E. coli	128	16	10	0.3	Negative
F4/3	E. coli	128	16	10	5	Negative
B4/6	E. coli	128	16	10	2.5	Negative
A4/11	E. coli	128	16	10	5	Negative
C4/3	E. coli	128	128	10	2.5	*merA*
E4/4	E. coli	128	128	10	2.5	Negative
D4/12	E. coli	128	16	10	2.5	*merA*
C4/12	E. coli	128	64	10	2.5	*merA*
G4/12	E. coli	128	16	10	2.5	Negative
I4/9	E. coli	128	128	10	2.5	*merA*
I4/3	E. coli	128	16	10	0.3	Negative
I4/13	E. coli	128	16	5	2.5	*merA*, *pcoA*, *silC*
H4/5	E. coli	128	16	10	0.3	Negative
H6/20	Salmonella spp.	128	128	10	0.15	Negative
G6/9	Salmonella spp.	128	16	10	0.625	*merA*, *pcoA*, *silC*
G6/13	Salmonella spp.	128	64	10	0.15	*merA*, *silC*
I2/5	Enterobacter spp.	128	128	10	20	*pcoA*, *silC*
I2/2	Enterobacter spp.	128	128	10	20	*pcoA*, *silC*
F2/6	Enterobacter spp.	128	128	0.625	0.15	*merA*
B1/10	P. stuanti	128	128	10	20	*merA*
Susceptible (*n* = 20)						
Kpff160	K. pneumoniae	128	128	10	10	*arsA*, *merA*, *pcoA*, *silC*
Kpff217	K. pneumoniae	128	16	10	0.3	*pcoA*, *silC*
KpFF11	K. pneumoniae	128	128	10	5	*arsA*, *merA*, *pcoA*, *silC*
KpFF197	K. pneumoniae	128	16	10	0.625	*silC*
KpFF177	K. pneumoniae	128	16	10	0.3	*pcoA*
KpFF296	K. pneumoniae	128	16	10	10	*arsA*, *pcoA*, *silC*
KpFF101	K. pneumoniae	256	16	10	10	Negative
KpFF264	K. pneumoniae	128	16	10	0.15	Negative
KpFF267	K. pneumoniae	128	16	10	0.15	Negative
KpFF153	K. pneumoniae	128	16	10	0.3	*pcoA*
Ec66	E. coli	128	8	10	0.15	Negative
Ec9	E. coli	128	16	10	0.15	Negative
Ec63	E. coli	128	8	10	0.15	Negative
Ec59	E. coli	128	8	5	0.15	Negative
Ec60	E. coli	128	16	5	0.15	Negative
Ec166	E. coli	128	8	10	0.15	Negative
Ec284	E. coli	128	8	10	0.625	Negative
Ec61	E. coli	128	128	10	5	Negative
Ec141	E. coli	128	16	10	0.15	Negative
Ec98	E. coli	128	16	10	0.15	Negative
Transconjugants and controls						
25922	E. coli	64	16	5	0.15	Negative
GFP	E. coli	64	16	5	1.25	Negative
TCE5/19	E. coli	64	16	5	2.5	*pcoA*
TCN12	E. coli	128	64	5	10	*arsA*, *pcoA*, *merA*
TCN22	E. coli	128	8	5	2.5	*pcoA*

**FIG 1:**
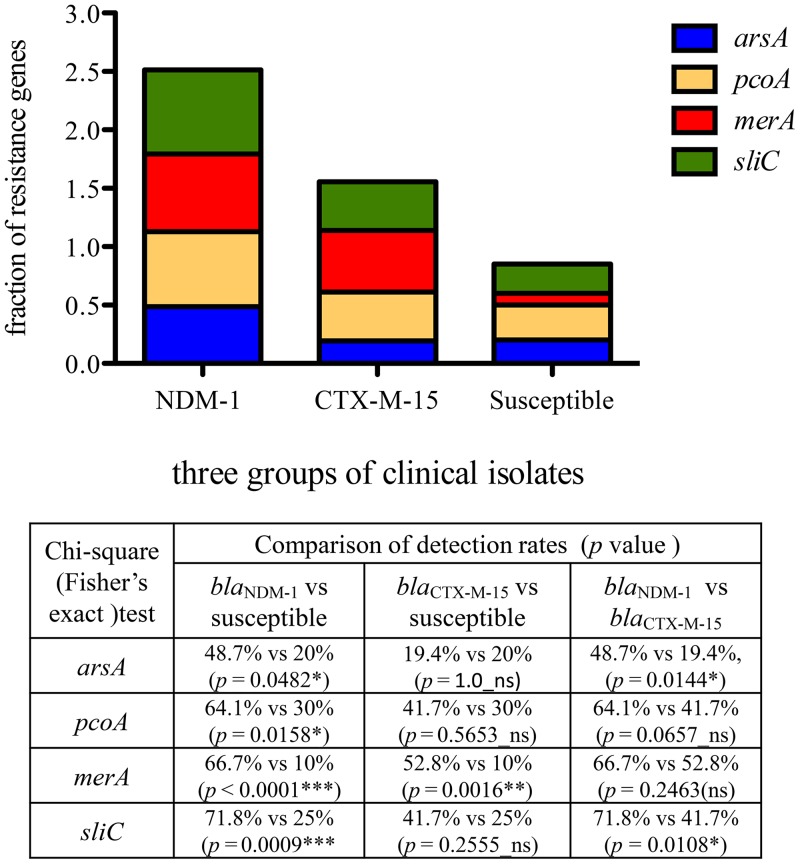
Occurrence of heavy metal resistance genes in 95 clinical isolates. *P* values were calculated using chi-square and Fisher's exact tests. *, 0.01 < *P* ≤ 0.05; **, 0.001 < *P* ≤ 0.01; ***, *P* ≤ 0.001. ns, not significant.

The presence of four heavy metal resistance genes was confirmed by PCR: *merA* for Hg^2+^, *arsA* for As^3+^, *pcoA* for Cu^2+^, and *silC* for Ag^+^. Primers were designed by primer 3 (Geneious Pro 5.5.6) and the NCBI primer designing tool (https://www.ncbi.nlm.nih.gov/tools/primer-blast/) (Table 2). PCRs were performed under the following conditions: initial denaturation at 95°C for 5 min, followed by 30 cycles of denaturation at 95°C for 45 s, annealing at 58°C to 60°C for 45 s and extension at 72°C for 45 s, and final extension at 72°C for 5 min. The purified PCR products were randomly selected for following sequencing analyses (Eurofins Genomics, Germany). The *silC*, *merA*, *pcoA*, and *arsA* genes were dispersed throughout our *bla*_NDM-1_-positive isolates, with 28/39 (71.8%), 26/39 (66.7%), 25/39 (64.1%), and 19/39 (48.7%), respectively (Fig. 1). Similarly, in *bla*_CTX-M-15_-producing isolates, the most prevalent heavy metal resistance gene was *merA* (19/36 [52.8%]). The genes *arsA*, *pcoA*, and *silC* were only detected in 7 (19.4%), 15 (41.7%), and 15 (41.7%) isolates, respectively. In contrast, the relatively low prevalences of *pcoA*, *silC*, *arsA*, and *merA* genes were identified in susceptible isolates, with detection rates of 30.0% (6/20), 25.0% (5/20), 20% (4/20), and 10% (2/20), respectively (Fig. 1). In addition, statistical comparisons with these metal resistance genes in three groups of isolates were conducted using chi-square and Fisher's exact tests, where a *P* value of ≤0.05 was considered significant. The prevalences of *silC* (71.8% versus 25.0%; *P* = 0.0009), *merA* (66.7% versus 10.0%; *P* < 0.0001), *pcoA* (64.1% versus 30.0%; *P* = 0.0158), and *arsA* (48.7% versus 20.0%; *P* = 0.0482) genes detected in *bla*_NDM-1_-positive isolates were all markedly higher than those in susceptible isolates. Furthermore, the detection rates of *silC* (71.8% versus 41.7%; *P* = 0.0108) and *arsA* (48.7% versus 19.4%; *P* = 0.0144) in *bla*_NDM-1_-positive isolates were significantly higher than those in *bla*_CTX-M-15_- producing isolates (Fig. 1).

**TABLE 2:** Details of primers used for heavy metal resistance gene detection in this study

Metal ion	Primer	Sequence (5′→3′)	Temperature (°C)	Size (bp)	GenBank accession no. or GeneID
Hg^2+^	*merA_*F1	CTGCGCCGGGAAAGTCCGTT	58	1,035	DQ126685
	*merA_*R1	GCCGATGAGCCGTCCGCTAC			
	*merA_*F2	GAGCTTCAACCCTTCGACCA	60	849	575669924
	*merA_*R2	AGCGAGACGATTCCTAAGCG			
As^3+^	*arsA_*F1	CAGTACCGACCCGGCCTCCA	58	861	CP000648
	*arsA_*R1	AGGCCGTGTTCACTGCGAGC			
	*arsA_*F2	GGCTGGAAAAACAGCGTGAG	58	1,002	387605479
	*arsA_*R2	CCTGCAAATTAGCCGCTTCC			
Cu^2+^	*pcoA_*F	CGGCCAGGTTCACGTCCGTC	58	1,371	NC_009649
	*pcoA_*R	TGCCAGTTGCCGCATCCCTG			
Ag^+^	*silC_*F1	CGTAGCGCAAGCGTGTCGGA	58	1,090	NC_009649
	*silC_*R1	ATATCAGCGGCCCGCAGCAC			
	*silC_*F2	TTCAACGTCACGGATGCAGA	60	872	157412014
	*silC_*R2	AGCGTGTCGGAAACATCCTT			

**FIG 2:**
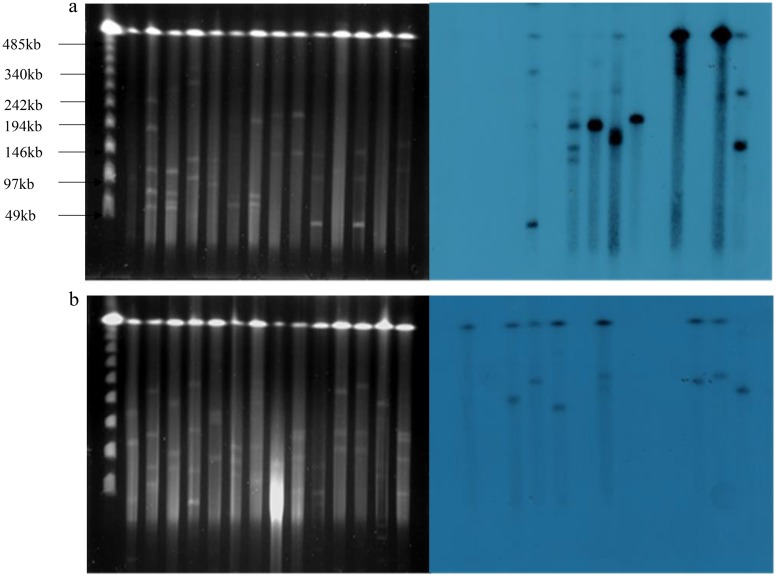
PFGE analysis of *bla*_NDM-1_-positive strains digested with S1 nuclease and hybridization with the *pcoA* gene probe (a) and *silC* gene probe (b). (a) Isolate order of lanes 1 to 14: N1, N2, N3, N4, N5, N6, N7, N8, N9, N10, N11, N12, N13, and N14. (b) Isolate order of lanes 1 to 14: N16, N17, N18, N19, N20, N21, N22, N23, 3, 26, N27, N28, N29, N31.

Previous studies have proposed the role of plasmids in conferring resistance to both antibiotics and heavy metals (7, 16, 17). In this study, the locations of the *pcoA*, *merA*, *silC*, and *arsA* genes were analyzed by pulsed-field gel electrophoresis with S1 nuclease (S1-PFGE) (Invitrogen Abingdon, UK). In brief, isolates carrying heavy metal resistance genes were randomly selected, and genomic DNA in agarose blocks was digested with S1 nuclease and probed. In-gel hybridization was performed with *pcoA*, *merA*, *silC*, and *arsA* gene probes labeled with ^32^P with a random primer method (Stratagene, Amsterdam, Netherlands). The results showed that *pcoA*, *merA*, *silC*, and *arsA* genes are located on a diverse range of plasmid backbones, differing from 50 to 500 kb in size (Fig. 2; see also Fig. S1 in the supplemental material). Heavy metal resistance genes were carried on more than one plasmid in many strains, and chromosomally located genes were identified (Fig. 2 and Fig. S1), suggesting significant plasticity.

Conjugation experiments were performed, as described previously (13), to investigate cotransfer of heavy metal and antibiotic resistance genes. Conjugations were performed with *bla*_NDM-1_- and *bla*_CTX-M-15_-positive donors with the rifampin-resistant recipient E. coli UAB190. Selection of *bla*_CTX-M-15_-positive transconjugants was performed on Brilliance UTI clarity agar (Oxoid, Ltd., Basingstoke, UK) supplemented with rifampin 100 mg/liter (Sigma-Aldrich, St. Louis, MO, USA) and cefotaxime 2 mg/liter. *bla*_NDM-1_-positive transconjugants were selected using rifampin with meropenem 0.5 mg/liter (AstraZeneca, London, UK). PCR for *bla*_NDM-1_ and *bla*_CTX-M-15_ genes was used for further confirmation of gene transfer (13, 18). Plasmid incompatibility groups were characterized by PCR-based replicon typing as previously described (19). A total of 18 and 14 transconjugants were obtained in E. coli UAB190 from 39 *bla*_NDM-1_ and 36 *bla*_CTX-M-15_ isolates, respectively. In 11 of 18 transconjugants, *bla*_NDM-1_ was located on IncA/C-type plasmids; 78.6% (11/14) of plasmids carrying *bla*_CTX-M-15_ belonged to IncFII, reflective of global molecular epidemiology (2, 20). Plasmids carrying *bla*_NDM-1_ from 6 transconjugants could not be typed. The heavy metal resistance genes *arsA*, *merA*, and *pcoA* were found on 2 *bla*_NDM-1_- and 1 *bla*_CTX-M-15_-positive plasmids, respectively (Table 1).

Our data indicate the abundance and mobility of heavy metal resistance genes (*pcoA*, *merA*, *silC*, and *arsA*) that can contribute to antibiotic-resistant gene dissemination and maintenance. Furthermore, many of these genes are found on transmissible plasmids. Therefore, our findings suggest that the coselection of heavy metal resistance genes in *bla*_NDM-1_- and *bla*_CTX-M-15_-positive isolates has significant implications for hospital and environmental (industrial waste) contamination with heavy metals.

## Supplementary Material

Supplemental material
