# Supplementary material for: Heavy Metal Resistance Genes Are Associated with blaNDM-1- and blaCTX-M-15-Carrying Enterobacteriaceae
Source: Antimicrob Agents Chemother. 2018 Apr 26;62(5):e02642-17. doi: 10.1128/AAC.02642-17 (PMC5923091; doi:10.1128/AAC.02642-17)
Supplement: Supplemental material [file supp_62_5_e02642-17__index.html]

Supplemental material 

# Heavy Metal Resistance Genes Are Associated with *bla*NDM-1- and *bla*CTX-M-15-Carrying Enterobacteriaceae

## Supplemental material

- Supplemental file 1 -

  Supplemental Figure S1

  PDF, 136K
