# Supplementary material for: Heavy Metal Resistance Genes Are Associated with blaNDM-1- and blaCTX-M-15-Carrying Enterobacteriaceae
Source: Antimicrob Agents Chemother. 2018 Apr 26;62(5):e02642-17. doi: 10.1128/AAC.02642-17 (PMC5923091; doi:10.1128/AAC.02642-17)
Supplement: Supplemental material [file AAC.02642-17_zac005187139s1.pdf]

Supplementary figure 1

a

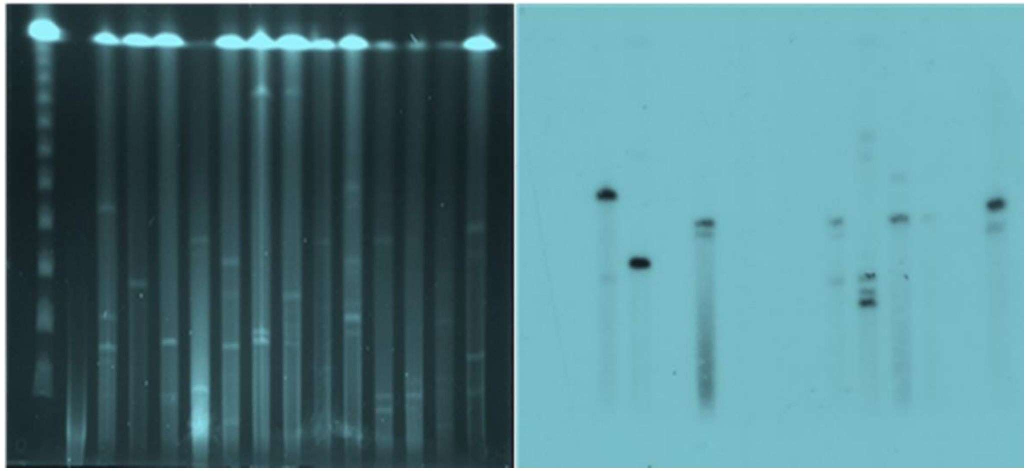

b

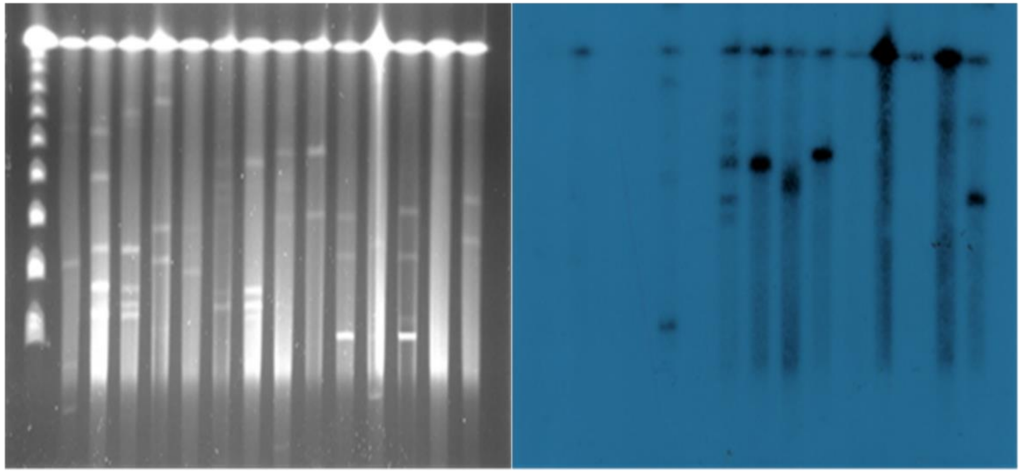

c

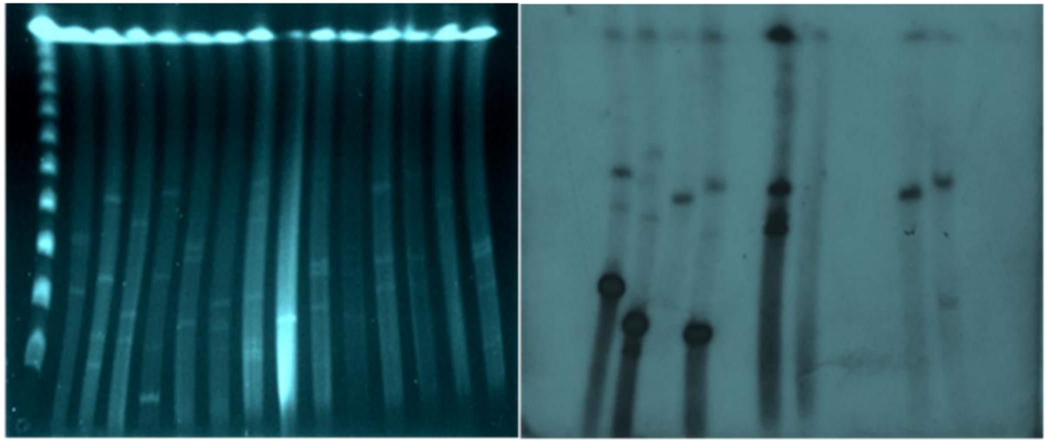

Supplementary Fig.1 PFGE analysis of *bla*$_{NDM-1}$-positive or *bla*$_{CTX-M-15}$-positive strains digested with S1 nuclease, and hybridization with *pcoA* gene probe (A), *silC* gene probe (B) and *arsA* gene probe (C), respectively.

Isolates order of lanes 1-14 in a: A5/3; A5/7; A5/4; C5/8; C5/7; C5/5; D5/12; D5/4; E5/14; E5/17; G5/2; G5/6; G5/11;K15.

Isolates order of lanes 1-14 in b: N1; N2; N3; N4; N6; N7; N8; N9; N10; N11; N12; N13; N14; N15.

Isolates order of lanes 1-14 in c: N15, N16, N17, N18, N19, N20, N21, N22, N23, N23, N26, N27, N28 and N29.
